# A Low-Cost Sensing System for Cooperative Air Quality Monitoring in Urban Areas

**DOI:** 10.3390/s150612242

**Published:** 2015-05-26

**Authors:** Simone Brienza, Andrea Galli, Giuseppe Anastasi, Paolo Bruschi

**Affiliations:** 1 Department of Information Engineering, University of Pisa, Largo Lucio Lazzarino 1, 56122 Pisa, Italy; E-Mails: galli@andrea.si (A.G.); giuseppe.anastasi@unipi.it (G.A.); 2 Department of Information Engineering, University of Pisa, Via G. Caruso 16, 56122 Pisa, Italy; E-Mail: paolo.bruschi@unipi.it

**Keywords:** urban sensing, air quality monitoring, cooperative system, participatory sensing

## Abstract

Air quality in urban areas is a very important topic as it closely affects the health of citizens. Recent studies highlight that the exposure to polluted air can increase the incidence of diseases and deteriorate the quality of life. Hence, it is necessary to develop tools for real-time air quality monitoring, so as to allow appropriate and timely decisions. In this paper, we present *uSense*, a low-cost cooperative monitoring tool that allows knowing, in real-time, the concentrations of polluting gases in various areas of the city. Specifically, users monitor the areas of their interest by deploying low-cost and low-power sensor nodes. In addition, they can share the collected data following a social networking approach. *uSense* has been tested through an in-field experimentation performed in different areas of a city. The obtained results are in line with those provided by the local environmental control authority and show that *uSense* can be profitably used for air quality monitoring.

## 1. Introduction

Air quality is a major concern for the public health, the environment and, ultimately, the economy of all the industrialized countries. In the last years, Europe and USA have considerably reduced the emissions of several airborne pollutants [1,2] such as carbon monoxide (CO), benzene (C_6_H_6_), sulphur dioxide (SO_2_), and lead (Pb). Anyway, nitrogen dioxide (NO_2_), ozone (O_3_), particulate matter (PM), and some other organic compounds still represent a serious threat. In such context, the “Air Quality in Europe” report—published by the European Environment Agency (EEA) [3] in November 2014—provides a thorough overview of measures and policies adopted at European level to improve the air quality and reduce the impact of air pollution on public health and ecosystems. In addition, the report describes the effects of air pollution on health, climate, and ecosystems. As shown also in [4], poor air quality can, in fact, cause ill health and premature deaths, as well as damages to ecosystems, crops, and buildings. Obviously, the effects are more serious in urban areas, where the majority of the population lives.

For all these reasons, an accurate real-time monitoring of air quality in urban areas is essential to enable appropriate and timely public decisions and, ultimately, is extremely important for preserving the citizens’ health. However, it is often difficult for citizens to obtain pollution data. In fact, air quality is usually monitored through large and expensive sensing stations, installed at some strategic locations, and managed by public authorities. Therefore, the monitoring (although accurate) is limited to few specific areas and the gathered measurements are sometimes not available to citizens. On the other hand, people are very interested in knowing air quality conditions in places where they live and spend much of their time, such as the area where their home is located, the school of their kids, their working place, public gardens, *etc.*

To overcome these limitations, in this paper, we present *uSense*, a sensing system for cooperative air quality monitoring in urban areas. *uSense* relies on small-size low-cost sensor nodes (equipped with gas sensors such as O_3_, CO and NO_2_) that can be privately installed by citizens inside their properties. Sensor nodes are powered by long-lasting batteries and use WiFi for data transfer, which makes their deployment very flexible and allows to easily place them outdoors. Owners of sensor nodes can also share their measurements, through a social networking approach, thus enabling cooperative sensing. This allows a real-time and fine-grained air quality monitoring of urban areas. The proposed system has been implemented and tested through in-field experimentation. 

The main goal of our study is to investigate whether a low-cost monitoring system can provide reliable indications about air quality in a place and, hence, can be used in practice. To this end, we have compared the pollution measurements obtained by *uSense* with the limit values specified by law and with the official measurements made available by the local control authority. From our experimental study, it emerges that, even though measurements provided by low-cost sensors are not as accurate as official data, nevertheless they can provide useful information about air quality in a specific location.

The rest of the paper is organized as follows. Section 2 describes the previous solutions already present in the literature, remarking the improvements introduced by *uSense*. Section 3 provides an overall description of *uSense*. Section 4 introduces the concept of Air Quality Index and shows how it can be calculated. Section 5 discusses the system architecture, whereas Section 6 provides some implementation details. Section 7 shows the results obtained using *uSense* to monitor some urban areas for one month (May 2014). Finally, Section 8 concludes the paper.

## 2. Previous Solutions

Sharing data and information with other people is becoming more and more common, thanks to the increasing popularity of personal devices (e.g., smartphones) that integrate low-cost sensors and provide fast and reliable data connectivity. Following an approach known as *Participatory Sensing*, users can gather information of interest and make it accessible to other people. Generally, data shared by users—relative to their positions—are processed and aggregated. This way, the other users of the system can access more complete and reliable information. Due to this trend, in recent years we witnessed a proliferation of devices and applications aimed at collecting and sharing data. The areas of application are many, ranging from health and fitness to environmental monitoring, from transportation monitoring to urban sensing. Especially in urban areas, many solutions have been proposed in order to improve the quality of life.

For instance, in [5] the authors propose *Ear-Phone*, a system that makes use of the microphone mounted on smartphones (preliminarily calibrated) to produce noise maps of a city, so as to allow citizens to avoid the noisiest areas. 

Similarly, several solutions have been proposed in the literature in order to monitor airborne pollutants with mobile low-cost sensors and share the obtained measurements with other users. For instance, *InAir* [6] and *MAQS* [7] face the issue of indoor air quality sensing. Specifically, *InAir* relies on stationary gas sensors placed inside users’ homes. A dedicated display on each sensor node visualizes the air quality values measured in the room and the values acquired by the other nodes in the other parts of the house. *MAQS*, instead, considers a mobile wearable sensing system that provides air quality information for each room visited by the user. The obtained data can be shared, through smartphones, with people who do not carry the sensing system. 

However, our interest is mainly focused on outdoor sensing. Also in this case, wearable sensors have been used to monitor air quality, for instance in *CitiSense* [8] and *Common* *Sense* [9]. Both these solutions rely on small, battery-powered sensor nodes that measure the concentrations of polluting gases and send air quality data to users’ smartphones through Bluetooth. The obtained data and the GPS coordinates are then shared with other users through a dedicated website, an Android app or social networks. Obviously, the small size of the sensor nodes and the required connectivity highly affect the lifetime of the battery, which needs to be frequently recharged.

A similar approach is also followed in [10] where the authors propose *GasMobile*, a small and portable ozone measurement system. Essentially, it relies on a particular sensor equipped with a serial transmitter board that allows to directly connect the sensor to the user’s smartphone through a USB port. The system is extremely compact and leverages an Android application to calibrate the sensor and upload the data to an ad hoc server. However, the readings are very limited, since they refer just to a single gas. Extending the measurements to other substances would considerably complicate the device, increasing its size and shortening its lifetime. 

Conversely, in [11] the authors propose a solution to derive high-resolution air pollution maps for urban areas, using nodes provided with several sensors, such as UFP (ultrafine particles), CO, O_3_, and NO_2_ sensors. Basically, the node acquires a location information through its GPS receiver, then, it transmits the gathered pollution measurements via the cellular network (*i.e.*, GSM) to the back-end server for further processing. The authors also propose a novel modeling approach to create pollution maps with high spatial and temporal resolution starting from the obtained measurements. However, differently from *uSense*, nodes are installed on buses and cannot be moved, once deployed. Therefore, they cannot be directly managed by citizens. This is a major drawback, since a user could not be able to know the pollution level in the areas of her/his interest.

Compared to the previously presented solutions, *uSense* allows to monitor the concentration levels of several air pollutants (*i.e.*, Ozone O_3_, Carbon monoxide CO, and Nitrogen dioxide NO_2_), by using small low-cost sensor nodes. These nodes can be deployed by the users in their areas of interest and moved whenever they want to monitor a different place. In fact, the proposed solution leverages on long-lasting batteries, which do not need to be frequently recharged, and exploits WiFi for data transfer, so that nodes can be easily placed outside as well. The *uSense* solution presented in this paper extends a previous version proposed in [12]. With respect to it, a new formulation of the Air Quality Index has been considered (detailed in Section 4). Consequently, all the obtained results have been revised according to the new definition. In addition, the results have been compared with the official measurements published by the local environmental control agency.

## 3. System Overview 

Through *uSense*, a user can monitor the air quality near her/his house, just by placing a small sensor node in her/his property, for instance in a garden, a balcony, a window sill, or hung to an outside wall. Basically, the node periodically measures—through proper sensors—the concentrations of some airborne pollutants. Then, the obtained data are sent to the *uSense* database via the Internet, and are made accessible to all the other *uSense* users. This way, in a cooperative fashion, each user contributes to monitor part of the city. Clearly, the system is particularly useful in urban areas where many sensor nodes have been placed. In this case, in fact—since pollution data are shared—all the *uSense* users can know, in real time, the pollution level of the various areas of the city. In addition, they can focus on specific regions of interest (e.g., their homes, workplaces, kids’ schools, *etc.*) to visualize the pollution level in the current day, or in the past. Such information can be exploited by users in several ways. For instance, they can decide to reach a destination searching for the less polluted path, or avoid going out during the most polluted hours. 

In details, the actions performed by *uSense*, from the initial acquisition of data to their publication, are the following:

(1) *Gas Sampling.* In order to monitor the air quality inside an area of interest, a user has only to deploy one or more sensor nodes. These nodes measure the concentration in the air of some damaging pollutants, e.g., *O_3_ (Ozone), CO (Carbon monoxide),* and *NO_2_ (Nitrogen dioxide)*. The measurements are performed periodically (e.g., every 30 min).
(2) *Data Transfer.* After that the concentrations of the various pollutants have been measured, the obtained data are arranged in a packet and transmitted to the *uSense* server, through the Internet, using a wireless technology (e.g., WiFi or GPRS).
(3) *Air Quality Index Calculation.* Once the *uSense* server has received the gas concentrations from a sensor node, it calculates the *Air Quality Index (AQI)* for the area corresponding to the node. Essentially, the server derives a number that indicates how good/bad the air quality is, so that increasing values correspond to higher pollution levels. Formulas for AQI calculation are defined by government agencies and vary from country to country and, sometimes, also from region to region, inside the same country. For instance, the index used in the USA—defined by the Environmental Protection Agency—is presented in [13]. In Europe, a Common Air Quality Index [14] has been developed, so as to present the air quality situation in European cities in a comparable and easily understandable way. However, the adoption of this index is not compulsory for European countries. Therefore, at the present day, only few cities and regions use it, while each country maintains its own regulations and indices. Details about the *Air Quality Index* adopted in *uSense* are provided in the following section.
(4) *Data sharing and view.* Data obtained from sensor nodes are stored inside a database and are made accessible to the *uSense* community, following a collaborative paradigm. This way, each user can view the AQI and gas levels regarding her/his nodes as well as the other users’ nodes. In addition, users can simply access air quality data even if they do not participate in the monitoring process (*i.e*., they do not own sensor nodes). *uSense* provides several mechanisms for exporting and visualizing data. They can be accessed through a Web interface, a mobile application, or Web services and can be presented in many ways. For instance, users can view the position of sensor nodes in a map, they can view the pollution levels of the various areas of the city, or search for the less polluted paths, and so on. Further details are provided in Section 5.

In order to be practically used by users, *uSense* provides some additional features:

— *Easy placement.* Sensor nodes are small and can be easily moved from a place to another. Furthermore, they can be placed outdoors, since they are battery-powered and the data communication is wireless.
— *Easy installation and access. uSense* can be easily set up by users. In fact, sensor nodes require just few steps to be fully working (see below for details). In addition, each user can access the data produced by her/his sensor nodes (or by the other users’ ones), simply creating a personal account in *uSense*, through the Web interface.
— *Robustness.* Each sensor node is inserted in a box, aimed at protecting it from atmospheric agents and impacts. Moreover, in order to provide a reliable service, whenever the access point/router is temporarily not available (e.g., due to weather conditions, channel errors, connection problems, hardware crashes, or blackouts) data are provisionally stored in a SD card, until the wireless connection is available again.

## 4. Air Quality Index

In each country, air quality monitoring is regulated by law. For instance, in Europe, the air quality Directive 2008/50/EC [15] defines the limit values for each pollutant and specifies the methods for measuring pollutants’ concentrations. Obviously, low-cost gas sensors (as the ones used in *uSense*) do not always reach the quality objectives prescribed by the legislation. Indeed, *uSense* is not intended to provide official measurements. Instead, it aims to provide useful and easy-to-read indications about the air quality in specific locations, making people aware of air pollution in the area where they live. 

In this perspective, we have defined a simple Air Quality Index in order to provide an easily readable and understandable measure of air pollution level, even for non-experts. Basically, for each sensor node, *uSense* returns a number that synthetically represents the degree of pollution in the air. No specific knowledge is required to interpret this value. Simply, the higher the index value, the more the air is polluted. To calculate AQI, concentrations of several air pollutants are taken into account and compared to the limits fixed by law. 

In *uSense*, the AQI is obtained by comparing the average concentrations of the monitored gases, measured in the considered day, to the limit values specified by national regulations, as follows:

(1) $$AQI=\text{max }\left\{\frac{G_{1}^{meas}}{G_{1}^{lim}},\;\frac{G_{2}^{meas}}{G_{2}^{lim}},\;\frac{G_{3}^{meas}}{G_{3}^{lim}},\;\ldots ,\;\frac{G_{N}^{meas}}{G_{N}^{lim}}\right\}$$

Specifically, let us assume we are monitoring N polluting gases. For each considered gas $G_{i}$ (with i ranging from 1 to N), $G_{i}^{meas}$ is obtained from the measurements performed throughout the day, considering the averaging period specified by law, whereas $G_{i}^{lim}\;$indicates the allowed concentration limit. In Equation (1), the concentration of each considered pollutant is divided for its reference limit. Then, the AQI is chosen as the highest value (corresponding to the pollutant with the highest concentration) among the obtained ratios. This way, the index warns us if one or more pollutants have exceeded the maximum concentration permitted. 

In the current implementation of *uSense* we only monitor O_3_, NO_2_, and CO. Consequently, Equation (1) can be rewritten as:

(2) $$AQI=\text{max }\left\{\frac{O_{3}^{meas}}{O_{3}^{lim}},\;\frac{NO_{2}^{meas}}{NO_{2}^{lim}},\;\frac{CO^{meas}}{CO^{lim}}\right\}$$

Limit values and averaging periods for O_3_, NO_2_, and CO (as specified in [15]) are reported in Table 1.

**Table 1:** O_3_, NO_2_ and CO averaging periods and limit values.

Pollutant	Averaging Period	Limit
Ozone O_3_	maximum hourly average	180 µg/m^3^
Nitrogen dioxide NO_2_	maximum hourly average	200 µg/m^3^
Carbon monoxide CO	daily maximum 8-hour average	10 mg/m^3^

To make the AQI value easily interpretable by eye, we have considered five air quality classes and we have assigned a color to each one. The first two classes indicate that reference limits have not been exceeded and, thus, there are no problems related to air quality. The other three classes, instead, warn that—with different levels of severity—some gases have exceeded the limit fixed by law. Classes and colors are reported in Table 2. 

**Table 2:** Air Quality Index and Levels.

Air Quality Index	Air Quality Classes	Color
From 0 to 0.5	Good	
From 0.5 to 1	Fair	
From 1 to 1.5	Moderate	
From 1.5 to 2	Unhealthy	
More than 2	Insalubrious	

## 5. System Architecture

**Figure 1:**
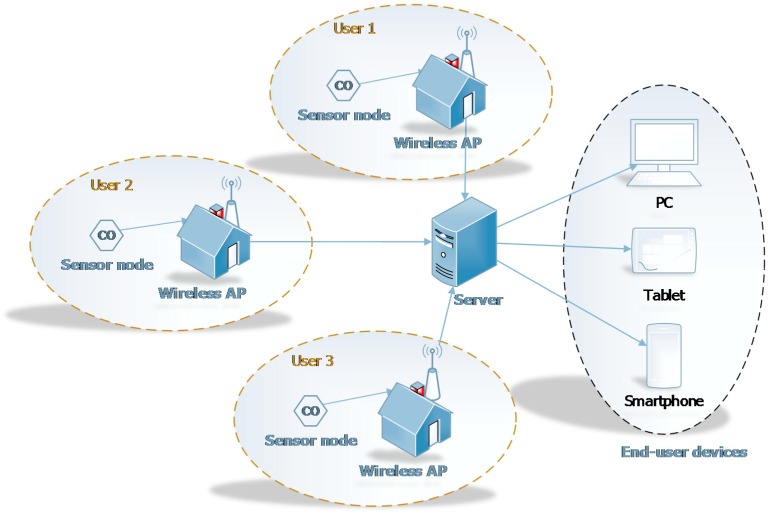
System architecture.

In this section, we present the architecture of *uSense*. As shown in Figure 1, *uSense* includes several components, *i.e.*, *sensor nodes*, *wireless access points*, *server* and *end-user* *devices*. They are described in the following subsections.

### 5.1. Sensor Nodes

*uSense* handles the measurements performed by a number of sensor nodes positioned at the users’ premises. Basically, sensor nodes measure the concentration of the airborne pollutants and send the acquired data to the *uSense* server. In order to extend their battery life, they use a duty-cycle mechanism that alternates activity and sleep periods. Specifically, each sensor node periodically (e.g., every 30 min) performs the following actions:

(1) *Sensor activation and sampling.* The gas sensors present on the gas board of the node are powered one at a time. After a warm-up period, the first sensor is read, providing a measurement for its correspondent gas. Then, it is switched off and another sensor is powered on (so as to avoid power consumptions peaks). The procedure is repeated for every gas sensor on the board, until all the measurements have been performed. This allows keeping the sensors on for the minimum time requested to take a measurement. The minimum warm-up period necessary to obtain a reliable measurement is indicated for each sensor in the corresponding datasheets. For all the sensors used in this work, we used a warm-up time of 30 s. The overall time required to read all the three sensors used, including the relative warm-up times and acquisition times, is much less than the considered sampling period (30 min).
(2) *Processing and transmission.* At this point, the obtained data are merged together to form a packet, containing also the univocal identifier of the node and a timestamp. Afterwards, the communication module is initialized. If the Internet connection is available, the sensor node contacts the *uSense* server and immediately sends the string, via *HTTP (HyperText Transfer Protocol)*. Otherwise, if no connection is available, the string is temporarily stored in the SD card (mounted by each node). The stored data will be transmitted to the server in the future, as soon as the connection is available again. This *opportunistic* approach for data communication allows the system to work correctly even when connectivity is intermittent (e.g., the user’s WiFi router may be switched off during certain periods).
(3) *Sleep.* Finally, the node disables the communication module and enters the low-power state. The only component that remains active is the real time clock (RTC), that wakes up the MCU *(MicroController Unit)*—through an interrupt—after a predefined time interval (e.g., 30 min). Then, steps 1–3 are performed again.

### 5.2. Wireless Access Points

After data have been acquired by sensor nodes, they are sent to the server through a wireless access point connected to the Internet. In principle, any wireless technology (e.g., WiFi, ZigBee, Bluetooth, GPRS) can be used. However, we only considered WiFi connectivity in our current implementation. Hence, the user is assumed to have a WiFi access point (or router) covering the area where the sensor node is located.

### 5.3. Server

The server part of the system consists of three different submodules that accomplish different tasks. 

*Database.* It stores information about users, sensor nodes, and measurements taken by sensors. Specifically, for each sensor node, the database contains its geographical coordinates (see below for details) and all the data regarding measured AQI values and gas concentrations. 

*Web Server.* It performs two different functions, *i.e.*, *data storage* and *data presentation* to users. First of all, it receives data from sensor nodes and stores them into the database. Specifically, whenever a sensor node has performed a measurement, the acquired data are sent to a dynamic page hosted by the Web server, using the GET method of the HTTP protocol. This page calculates the Air Quality Index and stores all the data in the database together with the identifier of the sensor node and the timestamp.

In addition, the Web server hosts the *uSense* website that allows users to access stored data and view the air quality levels. Specifically, through the Web interface, a user can

(a) create a personal account;
(b) associate sensor nodes to her/his account and set/modify their geographical coordinates (by simply locating them on a map);
(c) select a sensor node (even not belonging to the user) in a map and view the measurements performed in the current day or in a previous one;
(d) view a pollution map, where colored circles indicate the AQI level measured by each sensor (an example is provided in Figure 3);
(e) search for the less polluted path to reach a destination in the city.

*Application Server.* Data stored in the system are also accessible to third-party applications that can elaborate this information to provide additional features and/or statistics. To this end, *uSense* exports a number of Web services, following a *SOA (Service Oriented Architecture)* approach. The exported services are briefly described in Table 3.

**Table 3:** Exported services.

Service Name	Description
*getSensorDataService*	Given a node ID, returns the information contained in the database about that node
*getDatafromPeriodService*	Given a node ID and a range of dates, returns all the measurements performed by that node in the specified days
*getSensorRangeService*	Given a point (expressed as a pair of coordinates latitude-longitude) and a radius, returns the set of nodes placed in that area
*getPollutionDataService*	Given a point and a radius, returns the last measurements performed by nodes in that area

### 5.4. End-User Devices

Data stored in the system can be accessed by users in several ways. For instance, they can use the Web interface or Web services through a browser or third-party applications, respectively. Furthermore, we have implemented an *app* for smartphones, so as to allow users to quickly and easily access *uSense* even from their mobiles. In this way, users can access the system wherever a data connection is available. The mobile app supplies all the features provided by the Web interface. In addition—exploiting the GPS receiver typically available on smartphones—it is possible to view in a map the sensor nodes closest to the user and check the last measured AQI.

## 6. Implementation

In this section, we provide some implementation details about *uSense*.

### 6.1. Sensor Nodes

**Figure 2:**
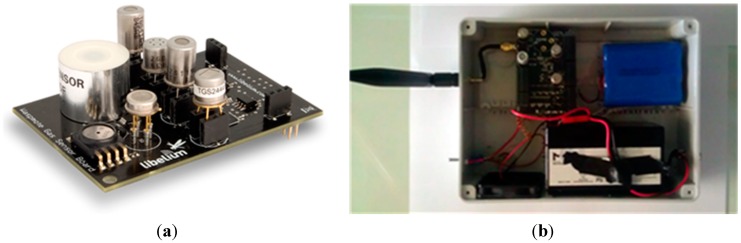
(**a**) Libelium Gas Sensor Board. (**b**) A sensor node inside its PVC box.

The sensor nodes used in *uSense* are *Libelium Waspmote* (Figure 2a)*.* They are equipped with an 8-bit microcontroller, and a WiFi module that allows TCP/IP and UDP/IP socket connections and HTTP/HTTPS communications. Each sensor node is provided with a gas sensor board, where CO, NO_2_, O_3_, temperature and humidity sensors have been mounted (specifically, *TGS2442*, *MiCS-2714* and *MiCS-2614* sensors for CO, NO_2_ and O_3_, respectively). In our prototype system, we have considered a sampling period of 30 min (*i.e.*, two samples per hour) for each gas under test. 

Waspmote nodes do not run any operating system. They can be programmed in *C* through a dedicated *IDE (Integrated Development Environment*), exploiting some libraries provided by the producer to interface with sensors, microcontroller and WiFi module. To allow easily deploying sensor nodes outside, we have realized a PVC box for each sensor node (shown in Figure 2b), containing—in addition to the Waspmote—an antenna, an activation button, a 6600 mAh rechargeable battery and a small brushless fan powered by an additional battery (so as to allow the air circulation). The use of a PVC housing may cause artifacts since this material can degrade in high concentrations of O_3_ and NO_2_, so that interaction between the gas molecules and the housing surface may be supposed. Indeed, this choice was motivated to minimize the cost of the single nodes, making future deployment of very dense monitoring systems economically sustainable. Use of highly inert plastics, such as *Polytetrafluoroethylene* *(PTFE)*, which would considerably increase the cost of individual nodes, will be considered for comparison purposes in future experiments. 

In order to obtain an acceptable accuracy, each sensor node should be individually calibrated. The calibration phase is often overlooked in articles regarding sensor networks. Unfortunately, a common characteristic of the miniaturized and inexpensive sensors that are typically mounted on sensor nodes is the large parameter tolerance. This means that the response curve of different samples of the same kind of sensor (e.g., NO_2_ sensor) prior calibration are so poorly matched that comparison of data produced by distinct sensor nodes are generally meaningless. For this reason, we preliminarily performed two-point calibration of all the sensors used in this work. Note that all the considered sensors are of resistive type and their responses are nearly linear in a log-log plot, as reported in datasheets. For this reason, the following expression was considered for the calculation of the gas concentration:

(3) $$log_{10}\left(C\right)=k_{1}log_{10}\left(R\right)+k_{2}$$

where *C* is the gas concentration, *R* the sensor resistance, whereas *k_1_* and *k_2_* are constants depending on the sensor type. The calibration procedure consisted in exposing the whole sensor nodes to different gas concentrations inside a small sealed chamber. The first step consisted in determining the optimal value of load resistance, which is a resistor placed in a series with the sensor resistance in order to form a voltage divider. The input voltage for the divider is a constant reference voltage, so that the output voltage is a function of the sensor resistance. An initial value for the load resistance was selected using the information provided by the sensor datasheets; this value is used to estimate the sensor resistance in the reference concentration. The final value of the load resistance is chosen in order to maximize the estimated output voltage swing across the operating concentration range. This is an important step, considering the relatively low-resolution AD converters (10 bit) that equip the sensor nodes. Tests were practically performed using two gas concentrations: (i) zero pollutant level, obtained exposing the sensor to synthetic air and (ii) reference level (*i.e.*, 1.45 ppm for NO_2_ and 100 ppm for CO) obtained by using gas cylinders, containing a known concentration of the pollutant in synthetic air. After setting a proper load resistance through direct programming of the sensor firmware, measurement of sensor resistance in the two concentration conditions allows to determine the constants *k_1_* and *k_2_* in Equation (3). Since a zero concentration is not representable with Equation (3), we have assumed that concentration in synthetic air is the one for which the sensor resistance reaches the value in pure air (reported in the datasheets). All measurements were taken after a delay of 30 s after switching on the sensors: this was necessary to allow the sensors to reach their operating temperature, as described in the sensor documentation. A gas mixture with precise O_3_ concentration was not available so that calibration of the O_3_ sensor was performed using only the synthetic air measurement (single-point calibration). 

The installation of a sensor node is quite straightforward. The user has only to digit the passphrase of her/his WiFi network inside a specific configuration file in the SD of the sensor node. Then, she/he has to create a personal account on the *uSense* website and associate the sensor node with the newly created account, by indicating where it is placed on a map. 

### 6.2. Server

The server part of the system is implemented as three distinct software processes (see Section 5.3). In particular, we used *PostgreSQL* as object-relational database management system. In addition, we resorted to *PostGIS*, a specific spatial extension of PostgreSQL, to handle geographical objects and queries (*i.e*., involving latitude and longitude coordinates).

**Figure 3:**
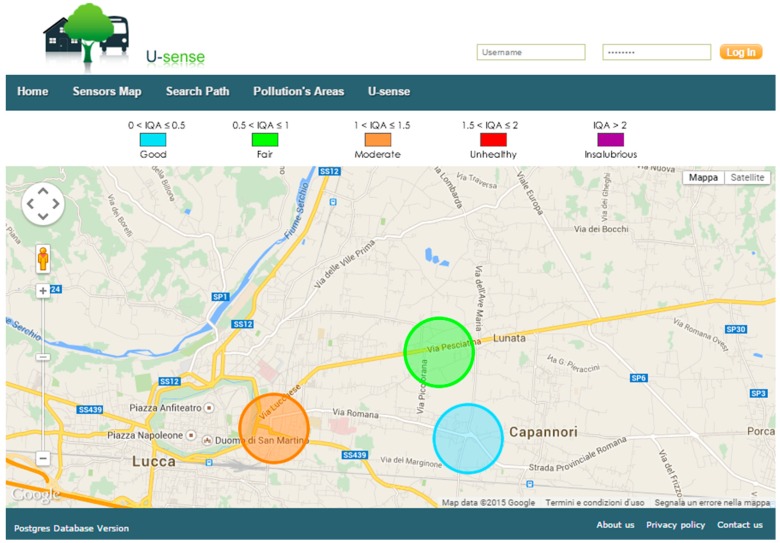
Webpage showing AQI levels for three sensor nodes.

The used Web server is *Apache* and the dynamic pages have been written in *PHP*. The website uses the *Google Maps API* to visualize sensor nodes and AQI levels on maps and determine the shortest paths between two points. Plots and graphs have been realized through the *Chart.js* JavaScript library. A screenshot of the website is shown in Figure 3. 

Finally, *JBoss* is the application server used to export Web services. Using JBoss—and, thus, *Java EE* (*Enterprise Edition)*—assures performance, scalability, security, and reliability. The web services are called through the *SOAP* protocol, over HTTP. The results of the implemented services are returned as *JSON* strings.

### 6.3. End-User Devices

**Figure 4:**
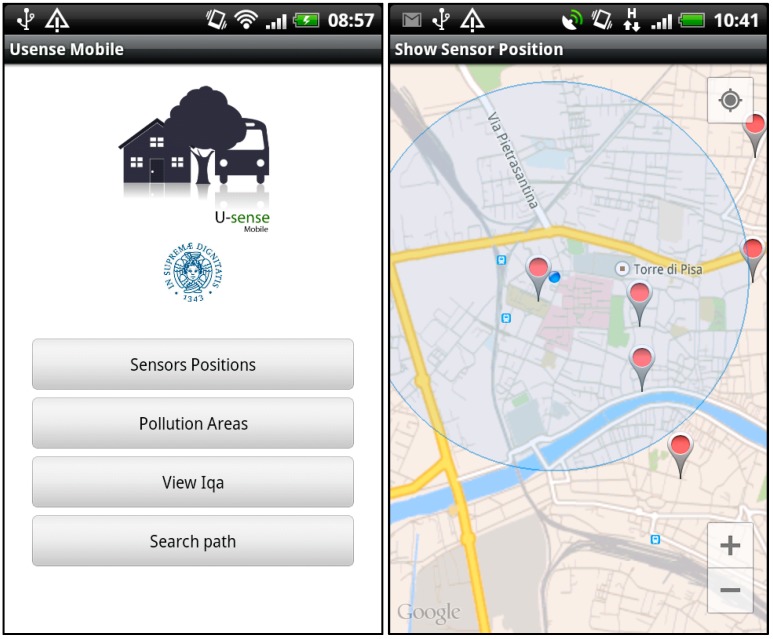
Mobile interface: initial page (**Left**) and nearest sensor nodes (**Right**).

The smartphone app has been realized for *Android*. Figure 4-left shows the initial page of the *uSense* app and the available choices. The screenshot in Figure 4-right shows the sensor nodes located near to the user. To implement this feature, we used the *Google Maps API* and the *GPS* receiver integrated in the smartphone.

## 7. Experimental Results

**Figure 5:**
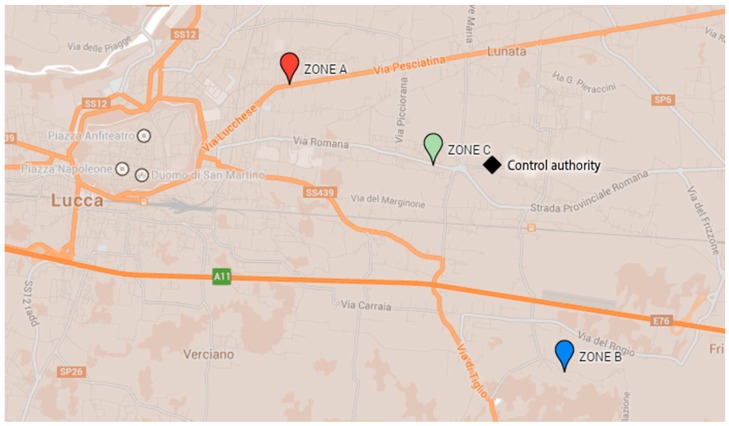
Locations of sensor nodes and control authority sensing station during in-field experimentation.

**Figure 6:**
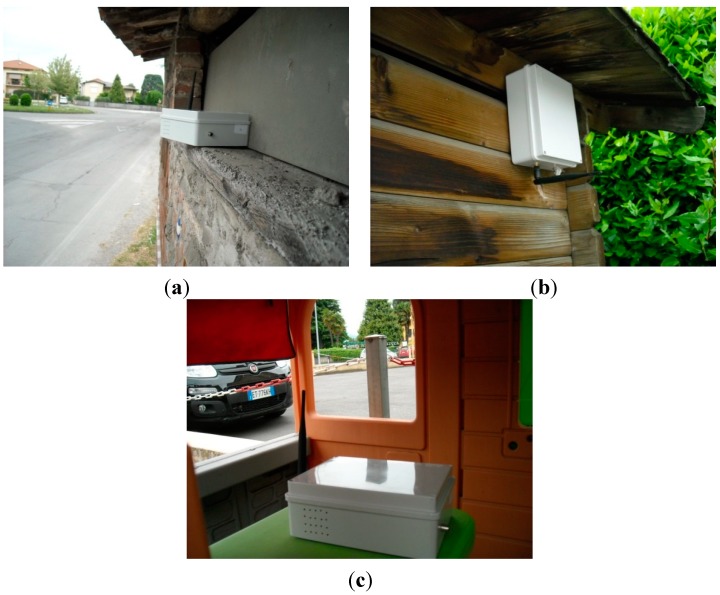
Sensor installation in Zone A (**a**), Zone B (**b**), Zone B (**c**).

In order to test the overall system, we performed an in-field experimentation of *uSense*. To this end, we installed a number of sensor nodes in different urban areas. In the following, we will refer to the experimental measurements obtained from three sensor nodes installed in the area of Lucca—a city in Tuscany—in strategic location with different expected pollution levels. Sensor nodes—inside their PVC boxes—were installed near roads, in order to investigate the impact of traffic on air quality. Specifically, we considered three areas (namely *Zone A*, *Zone B*, *Zone C*) with different traffic conditions and, hence, different expected pollution levels. Zone A is near the center of the city and is characterized by heavy traffic. On the contrary, Zone B is in the countryside and the sensor node is located near a rural road. Finally, Zone C is a suburban area with medium traffic intensity. Figure 5 shows the positions on the map of the three sensor nodes during the in-field experimentation, whereas Figure 6 shows the sensor nodes inside their boxes, as they have been placed in the various zones.

**Figure 7:**
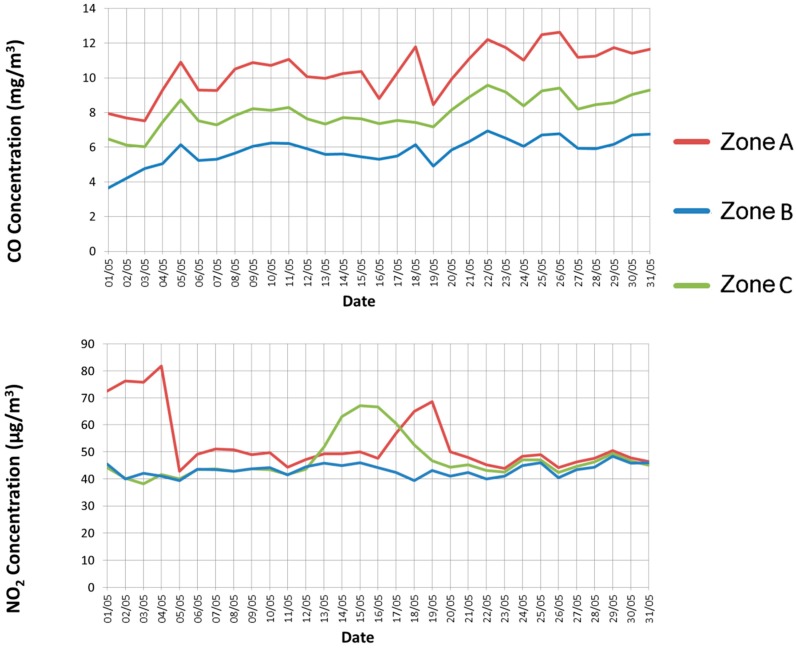
Experimental results: gas concentrations (NO_2_, and CO) in the three zones.

**Figure 8:**
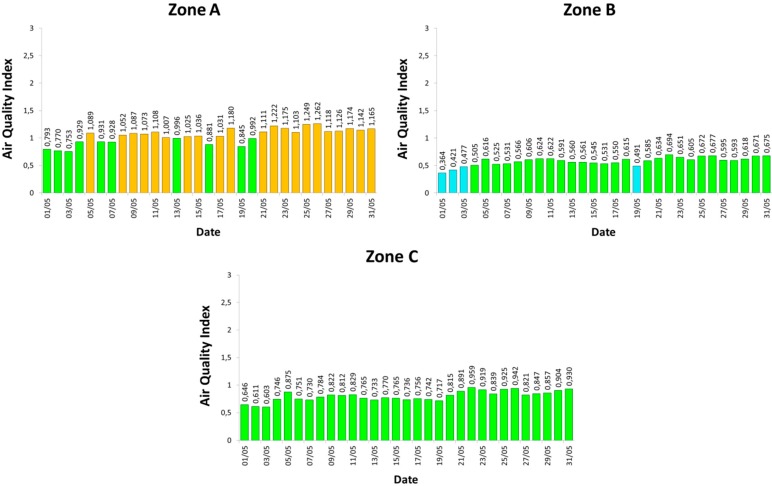
Experimental results: Air Quality Index (AQI) in the three zones.

The monitoring phase started on 1 May 2014 and ended on 1 June 2014. Since nodes were placed outdoors, the WiFi signal strength was quite variable, and, therefore, the WiFi connection was not optimal. However, we observed that no measurement was missed, thanks to the reliable data transfer service implemented in *uSense*. The experimental results are plotted in Figure 7 and Figure 8. 

Figure 7 shows, for each considered day, the maximum average concentration of CO, and NO_2_. In fact, these gases are the most significant ones to monitor the pollution due to traffic, since they are mainly produced, through combustion, by engines that run on fossil fuel, such as petroleum derivatives. Specifically, we show the daily maximum 8-h average value for CO and the maximum hourly average value for NO_2_ (similarly to how reference values are expressed in Italian regulations). Instead, Figure 8 reports the measured AQI values, according to the definition given in Section 4. The obtained results confirm our expectations. Zone A is the most polluted area, due to the transit of many vehicles, in particular trucks, and the lack of vegetation. Conversely, Zone B—a rural and low-traffic area—exhibits low pollution levels, which are absolutely negligible. Zone C, instead, shows intermediate gas concentrations, but which are still acceptable. 

**Figure 9:**
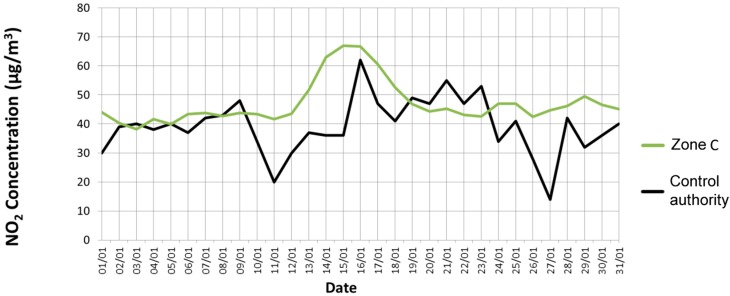
Experimental results: NO_2_ values measured in Zone C and values provided by the local environmental control authority.

To validate the collected results, we have compared them with the values measured by the local environmental control authority (namely ARPAT) for the entire period. All the data gathered by the environmental control authority are daily published and freely available in the ARPAT website [16]. Among the various monitoring stations of the control authority placed near the considered city, we have chosen the closest to the *uSense* sensor nodes. Specifically, we have compared the measurements obtained by the sensor node in Zone C with the ones provided by the ARPAT sensing station named LU-CAPANNORI (whose location is shown in Figure 5). The node in Zone C is distant less than 800 m from the sensing station of the control authority. Since the latter does not sample CO and O_3_, we have limited our comparison to NO_2_. As clearly emerges from Figure 9, our results match closely the values provided by the environmental control authority during the whole duration of the experiment.

## 8. Conclusions

In this paper, we have presented *uSense*, a low-cost sensing system for cooperative air quality monitoring in urban areas. The novel contribution of *uSense* is represented by the possibility for users to monitor the air pollution level inside their properties, by simply installing one or more sensor nodes. *uSense* follows a cooperative sensing approach, in the sense that the data collected by each sensor node are uploaded to a remote server and shared among the users of the system, so that each one can obtain a more complete and reliable information about the pollution of the various parts of her/his city. Data collected by *uSense* can be accessed in several ways, *i.e.*, through a Web Interface, a Mobile App or Web Services. 

Compared to other solutions present in the literature, *uSense* relies on small, low-power sensor nodes, provided with long-lasting rechargeable batteries (6600 mAh) and WiFi communication modules. This way, they can be easily moved and deployed outside, wherever there is WiFi coverage. For instance, they can be placed in a balcony, in a garden, on a window sill or can be hung to a wall. A sensor node can work even when the network connectivity is intermittent, so as to avoid data loss. In this case, in fact, the node can temporarily store the collected data in a SD card, waiting for the connection to be available again. In addition, given the modularity of the used *Libelium* board, extending the range of the monitored pollutant is straightforward.

*uSense* has been tested through an in-field experiment, in three different urban areas. The experimental measurements—compared with the values provided by the local environmental control agency—show that, even though measurements obtained from low-cost sensors are not as accurate as official data, they can still provide useful indications of air quality in a specific location. 

Also, it may be worthwhile emphasizing that the data quality provided by *uSense* depends on the accuracy of low-cost sensors. As the technology evolves, more accurate low-cost sensors will be made available and the data quality will improve accordingly. From this perspective, *uSense* can be considered an evolving low-cost monitoring platform whose data quality can benefit from developments in sensor technology.
